# Incidence of dementia among individuals 70 years and older in Norway: A HUNT study

**DOI:** 10.1177/13872877251371242

**Published:** 2025-08-29

**Authors:** Inger Molvik, Bjørn Heine Strand, Anne Marie Mork Rokstad, Eivind Aakhus, Stina Aam, Sverre Bergh, Anne Brækhus, Knut Engedal, Linda Gjøra, Grete Kjelvik, Marte Kvello-Alme, Gill Livingston, Fiona E Matthews, Karin Persson, Håvard Kjesbu Skjellegrind, Geir Selbæk

**Affiliations:** 1 The Norwegian National Centre for Ageing and Health, Vestfold Hospital Trust, Tønsberg, Norway; 2 Institute of Clinical Medicine, University of Oslo, Oslo, Norway; 3 Department of Geriatric Medicine, Oslo University Hospital, Oslo, Norway; 4 Norwegian Institute of Public Health, Norway of Public Health, Oslo, Norway; 5 Faculty of Health Sciences and Social Care, Molde University College, Molde, Norway; 6 Department of Geriatric Medicine, Clinic of Medicine, St Olavs hospital, Trondheim University Hospital, Trondheim, Norway; 7 Department of Neuromedicine and Movement Science, Faculty of Medicine and Health Science, NTNU-Norwegian University of Science and Technology, Trondheim, Norway; 8 The Research Centre for Age-related Functional Decline and Disease, Innlandet Hospital Trust, Ottestad, Norway; 9 Department of Neurology, Oslo University Hospital, Oslo, Norway; 10 Department of Psychiatry, Levanger Hospital, Nord-Trøndelag Hospital Trust, Levanger, Norway; 11 Department of Neurology, Nord-Trøndelag Hospital Trust, Levanger Hospital, Levanger, Norway; 12 Division of Psychiatry University College London, London, UK; 13 North London partnership trust NHS Foundation Trust, St Pancras Hospital, London, UK; 14 Institute for Clinical and Applied Health Research, University of Hull, Hull, UK; 15 HUNT Research Centre, Department of Public Health and Nursing, Faculty of Medicine and Health Sciences, Norwegian University of Science and Technology, Levanger, Norway; 16 Levanger Hospital, Nord-Trøndelag Hospital Trust, Levanger, Norway

**Keywords:** Alzheimer’s disease, dementia, epidemiology, incidence, population study

## Abstract

**Background:**

With increasing population longevity, Alzheimer's disease and dementia have become a health priority, and high-quality incidence estimates are needed.

**Objective:**

To provide reliable and precise incidence estimates of dementia applying a population-based sample of individuals aged 70+.

**Methods:**

A longitudinal cohort design was used, with baseline assessment in the Norwegian HUNT4 70+ study (2017–19) and at follow-up four years later (2021–23). Age-specific dementia incidence rates, standardized for the Norwegian population, were calculated as the number of new dementia cases per 1000 person-years assuming onset midway between study waves with inverse probability weights based on baseline factors associated with non-participation or death.

**Results:**

Among 5229 dementia-free individuals at baseline, 749 developed dementia over a 4.2-year period, resulting in a cumulative incidence proportion of 14.3%. At follow-up, 33.8% of new dementia cases showed no baseline cognitive impairment, while the rest had mild cognitive impairment (MCI). Of those with baseline MCI, 25.5% reverted to normal cognition, 48.2% remained MCI, and 26.2% developed dementia. The dementia incidence per 1000 person-years, was 43.9 (95% confidence interval (CI) 40.8, 47.1) (weighted for non-response and standardized to the dementia-free Norwegian population).

**Conclusions:**

Our study found higher dementia incidence rates in the 70+ population than hospital records indicate. Most individuals had preceding MCI, with similar numbers reverting to normal cognition as developing dementia. The projected incident dementia cases suggest a near doubling between 2023 and 2050, significantly impacting families and healthcare planning, including early detection and interventions.

## Introduction

Dementia is a progressive syndrome that impairs cognitive functioning, affect behavior, and the ability to carry out daily activities.^1^ Currently, 55 million individuals worldwide live with dementia, with numbers expected to reach 131.5 million by 2050.^2^ In Norway, the current dementia prevalence of 101,000, is projected to more than double by 2050.^3^ The impact of dementia extends beyond the individuals themselves, causing distress for their families, informal and formal caregivers. In 2018, the global cost of dementia care reached US$ 1 trillion.^4^ In Norway, dementia is estimated to be the most expensive among 144 health conditions.^5^ The World Health Organization (WHO) has identified dementia as a significant public health concern because of the profound social and economic implications.^6^ Although the ageing population is contributing to the growing number of people living with dementia, there is evidence suggesting a decrease in age-standardized prevalence and incidence of dementia in Europe and US,^7–11^ as well as Norway between 2000 and 2019.^12^ However, a recent study reported a higher prevalence in Norway in comparison to similar countries.^3^ In Norway, like other high-income countries, the population is ageing, and it is crucial to assess prevalence and incidence of dementia among older adults to understand current situation and predict future healthcare needs. The Trøndelag Health Study (HUNT),^13^ is a Norwegian population-based longitudinal study. The fourth wave of the study, referred to as HUNT4, was conducted from 2017 to 2019. Within this wave, a sub-study, HUNT4 70+, focused on individuals aged 70 and older. Participants from the HUNT4 70+ sub-study were re-evaluated four years later, which offers a unique opportunity to investigate the incidence of dementia and progression from MCI to dementia within this representative cohort. Our aim was to provide insights into current and future incidence estimates of dementia and conversion from MCI to dementia in the population-based HUNT study of individuals aged 70+.

## Methods

### Study population

This is a prospective longitudinal cohort study recruiting 5229 dementia free participants ages 70+ who had participated in HUNT4 70+ in 2017–19. These participants were part of the Ageing in Trøndelag study (AiT) in 2021–23, after a mean follow-up time of 4.2 years (range 2.9–5.2).

**Figure 1.**
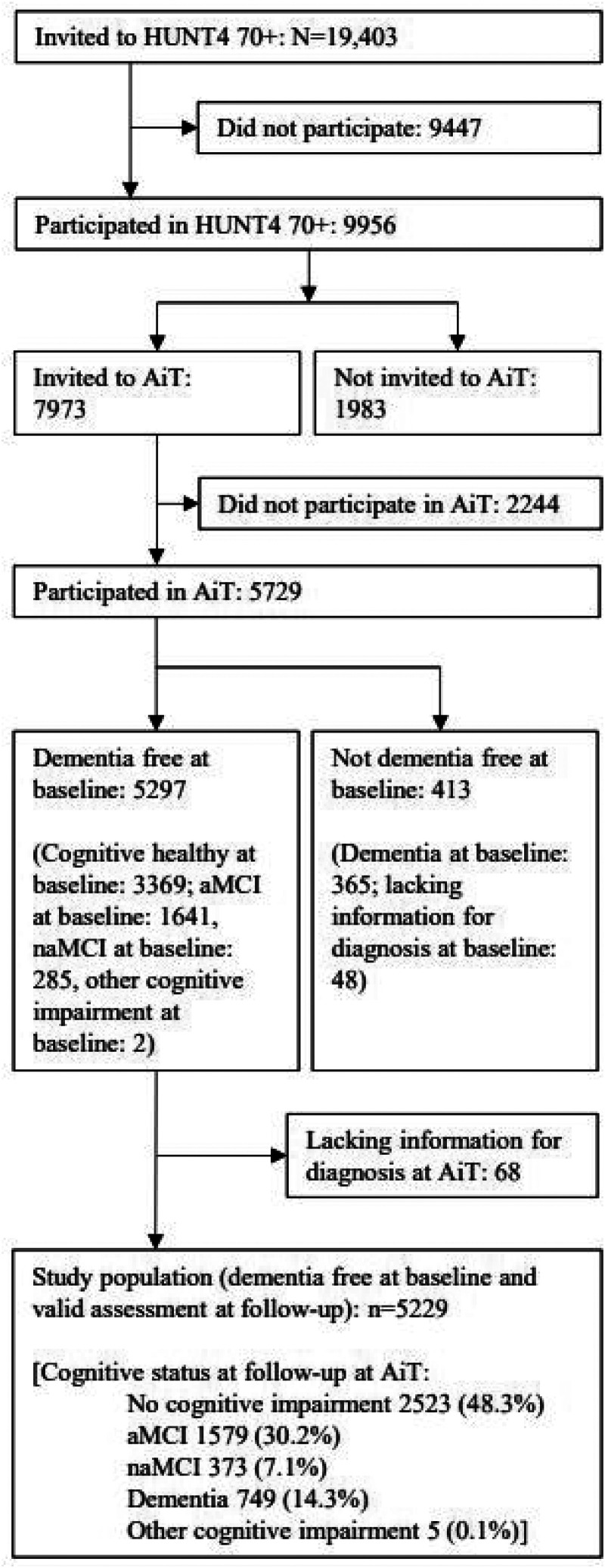
Flow-chart of progress through study.

In HUNT4 70+, all inhabitants aged 70+ were invited (N = 19,403), with 9956 (51.3%) participating (Figure 1). Those still alive and eligible (n = 7973) were invited to AiT, of whom 5729 (71.9%) participated. A total of 5229 were dementia-free at baseline (HUNT4 70+) and had valid assessments at follow-up (AiT). All residents aged 70 and older in the catchment area received an invitation and an information letter about the HUNT4 study in general and the HUNT4 70+ study in particular. Those who did not wish to participate, were not asked to provide a reason for their non-participation.

### Setting

The northern Trøndelag region, where the HUNT Studies were conducted, comprises rural areas and small towns with populations under 25,000 inhabitants. This region has a lower percentage of immigrants and highly educated individuals than the national average,^14^ but has similar cause-specific mortality, overall health, unemployment, and disability insurance rates.

### Assessments

The assessments at AiT mirrored the HUNT4 70+ baseline assessment. Participants underwent clinical evaluations by skilled healthcare personnel at field stations, or at home/nursing homes if needed. Assessors received standardized two-day training on dementia assessment and Short Physical Performance Battery (SPPB). Uniform protocols were used at both field stations and homes, with adaptations in nursing homes to align with residents’ capabilities, relying more on healthcare staff for data collection. Participants who self-reported cognitive decline or scored below age-adjusted cut-offs were asked to consent to structured telephone interviews with a next of kin. These interviews gathered additional information on cognitive changes, functional levels, neuropsychiatric symptoms, and potential dementia symptoms (detailed in the Supplemental Material). To assess cognition and diagnose mild cognitive impairment (MCI) or dementia, evaluations included daily life functioning, subjective cognitive decline, (Instrumental Activities of Daily Living Scale (I-ADL), Physical Self-Maintenance Scale (PSMS)),^15^ neuropsychiatric symptoms (Neuropsychiatric Inventory Questionnaire (NPI-Q)),^16^ presence of depression (Hospital Anxiety and Depression Scale (HADS)),^17^ and level of cognitive and functional impairment (Clinical Dementia Rating Scale (CDR)).^18^ Data on living arrangements, marital status, and education (primary (<10 years), secondary (10–12 years), tertiary (>12 years)) were also used. The cognitive assessment protocol included the Montreal Cognitive Assessment (MoCA),^19^ and the CERAD Word List Memory Task (WLMT),^20^ was assessed for individuals scoring ≥22 on MoCA. For nursing home residents, an adapted interview protocol was implemented. Knowledgeable healthcare personnel familiar with the participants provided insights into their cognitive functioning and dementia-related symptoms. In HUNT4 70+, when participants were deemed to have moderate to severe dementia based on information from health personnel with close knowledge of the participant, the Severe Impairment Battery-8 (SIB-8)^21^ was utilized instead of MoCA. SIB-8 is a condensed version of the Severe Impairment Battery,^22^ a well-validated instrument designed specifically for assessing cognition in individuals with moderate to severe dementia.

### Diagnosis of dementia or mild cognitive impairment

Dementia status in HUNT4 70+ and AiT was established using a consensus-based evaluation by two experts,^3^ and in cases where consensus was not reached, a third expert was consulted. A diagnostic expert group comprising eight medical doctors with extensive scientific and clinical expertise in geriatrics, geriatric psychiatry, or neurology, independently decided on diagnoses of dementia subtypes and mild cognitive impairment using DSM-5 diagnostic criteria.^23^ The diagnostic expert group had access to all available data from the AiT assessments. The expert group largely consisted of the same experts involved in the diagnostic processes for both HUNT4 70+ and AiT. Before initiating the diagnostic process, the experts participated in a seminar wherein diagnostic rules, including the diagnostic criteria, were thoroughly reviewed and a consensus was reached. In preparation for the diagnostic assessments, the experts independently classified 55 cases into one of the following categories: no cognitive impairment, mild cognitive impairment, dementia, or “could not be classified” based on the DSM-5 criteria.^23^ In a reliability study with all raters scoring a group of 55 cases selected to reflect complicated cases, the Fleiss’ kappa coefficient was 0.67 (95% CI 0.64, 0.70). For dementia alone, it was 0.75 (95% CI 0.70, 0.80). Subsequently, these results were discussed to harmonize the classifications among the experts. Standard diagnostic criteria from the DSM-5 were applied for MCI (mild neurocognitive disorder) and dementia (major neurocognitive disorder). MCI was further classified based on the predominant cognitive symptom: amnestic MCI (aMCI) involved primarily memory impairment, while non-amnestic MCI (naMCI) involved impairments in executive function, language, or visuospatial abilities with relatively spared memory function.^3,24^

### Statistical methods

Stata 18 was used for analyses. Not all baseline participants took part in the AiT follow-up, either due to death or other circumstances. The dropout was informative; it is likely that individuals more prone to developing dementia by the time of AiT were among those who dropped out, resulting in a healthy selection bias. Participants less likely to attend follow-up were typically men, older individuals, those with lower education levels, unmarried status, lower MoCA scores, poorer health, or lower SPPB scores. More specifically, in a logistic regression model with participation (yes = 1/no = 0) as the outcome and sex, age, education (compulsory, secondary tertiary), marital status, HADS ≥8 versus <8), MoCA (<22 versus ≥22), SPPB (<9 versus ≥9) as predictor variables, the OR for participation was 0.8 for men versus women; with age 70–74 as ref OR = 0.85 for age 75–79 and OR = 0.67 for age 80–84 and OR = 0.42 for age 85+; with compulsory education as ref OR = 1.21 for secondary and OR = 1.62 for tertiary education; married had OR = 1.25 compared to non-married; elevated HADS had OR = 0.92 (non-significant); poor MoCA had OR = 0.69; poor SPPB had OR = 0.59. All ORs had p < 0.001 if not stated otherwise. To account for this selection bias, inverse probability weights were calculated based on these baseline characteristics. Specifically, an indicator variable of participation in AiT (yes/no) was constructed. A logistic regression model was then fitted using this indicator variable as the dependent variable and the variables sex, age (70–74, 75–79, 80–84, 85+), education (primary, secondary, tertiary), marital status (married, non-married), depression (HADS-D < 8, ≥ 8), MoCA-score (1–21, 22–30) and SPPB score (0–8, 9–12) as independent variables. Due to possible informative missing, separate missing categories were included (sex and age had no missing). The probability of participating was estimated for all possible combinations of the independent variables. Inverse probability (IPW) weights were constructed. Weights ranged from 1.2–11.4, and was trimmed to an upper level of 5 (16 participants) to reduce impact of outliers.^25^ In addition, calibration weights were applied, to standardize the regional results to the full Norwegian population for year 2023 using the population sizes by age, sex, and education. This information was provided by microdata.no, a service from Statistics Norway. Dementia prevalence estimates from Gjøra et al.^3^ was used to estimate the dementia free population sizes by age and sex. These two sets of weights were applied to the dementia incidence estimates to account both for selective drop-out and to standardize the rates to the Norwegian population.

To further project the number of new dementia cases for the years 2030, 2040, and 2050, national population projections from Statistics Norway (SSB) were used (main alternative scenario). Based on age- and sex-specific dementia prevalence rates for five-year age groups over 70 years from the HUNT4 70+ study,^3^ the number of individuals without dementia at the beginning of each year was estimated. These dementia-free population estimates were then multiplied by age- and sex-specific dementia incidence rates to calculate the projected number of new dementia cases in the population without dementia for each respective year.

Dementia onset follows a steep age gradient, making it likely that onset occurs closer to the time of AiT. As such, using the midpoint between assessments as the default onset time may underestimate follow-up duration and thus bias incidence rates upward. However, a sensitivity analysis that included a later onset time estimate, at two-thirds of the interval rather than the midpoint, did not significantly change the findings of our study (the weighted and standardized dementia incidence rate per 1000 person years for the population aged 70+ was reduced from 43.9 to 41.1). This minor change did not affect our primary conclusions regarding dementia incidence in the studied population.

#### Dementia incidence proportion, and dementia incidence rate

The crude dementia incidence proportion was calculated as the cumulative percentage with dementia at AiT. Additionally, to take time into account, the dementia incidence rate was calculated as the number of participants developing dementia divided by 1000 person years. For participants who developed dementia, the exact transition time was unknown and assumed to occur midway between the two assessment waves.^26^ Weighted incidence was calculated using IPW. To allow study participants to change age group during follow-up, a Lexis-approach was applied using the *stsplit* command in Stata. Weighting was incorporated using the *pweights* in the *stset* command, where time in years between baseline assessment and follow-up assessment was used as time scale. The *strate* command was used to calculate incidence rates per 1000 person years, by age groups and sex with accompanying jackknife 95% confidence intervals. To account for drop-out and to standardize rates, we applied IPW and calibration weights simultaneously,^25^ using the Stata command (*svy*). To calculate IPW-weighted and standardized incidence rates, the total number of new cases divided by total person years were done using ratio command together with *svy* command in Stata, over age band (provided using *stsplit* command) and sex. In addition, for comparison purposes, the European standard population of 2013 and direct standardization was applied.^27^ To compare to studies with different age groups, we used identical age groups for standardized rates. For sensitivity analysis, we calculated standardized rates without IPW, and excluded nursing home population and home interviews performed at the (frail) respondents’ home.

## Results

In our study population (N = 5229), 53% were women. The female sample was on average 0.35 years older than the male sample (p < 0.01). Most of the study population was interviewed at field station at baseline (98.5%), with a few at institutions (0.2%, n = 9) or at home (1.4%, n = 72). Compared to those who remained dementia-free, those with dementia at follow-up were older, less educated, were more often single, depressed, and had lower baseline MoCA-scores (Table 1).

**Table 1.** Socio-demographic details of people in the study who were dementia free at HUNT4 70+. N = 5229.

	Cognitive diagnosis at follow-up at AiT							
	No cognitive impairment		MCI/other cognitive impairment		Dementia		Total	
Age, mean (SD)	75.4	(4.2)	75.5	(4.6)	78.6	(5.9)	75.9	(4.7)
MoCA HUNT4 70+, mean (SD)	25.6	(2.5)	23.6	(2.8)	21.4	(3.3)	24.3	(3.1)
MoCA at AiT, mean (SD)	25.7	(2.0)	22.1	(2.5)	16.2	(3.6)	23.0	(4.0)

HUNT4 70+ variables:	N	%	N	%	N	%	N	%
Total	2523	100	1957	100	749	100	5229	100
Women	1465	58%	919	47%	408	54%	2792	53%
Men	1058	42%	1038	53%	341	46%	2437	47%
Age								
70–74	1364	54%	1099	56%	244	33%	2707	52%
75–79	801	32%	529	27%	213	28%	1543	30%
80–84	279	11%	258	13%	159	21%	696	13%
85+	79	3%	71	4%	133	18%	283	5%
Education								
Basic	478	19%	469	24%	240	32%	1187	23%
Secondary	1093	43%	950	49%	338	45%	2381	46%
Tertiary	941	37%	517	26%	143	19%	1601	31%
Missing	11	0%	21	1%	28	4%	60	1%
Married								
No	754	30%	643	33%	302	40%	1699	32%
Yes	1758	70%	1305	67%	430	57%	3493	67%
Missing	11	0%	9	0%	17	2%	37	1%
Depression, (HADS-D) at HUNT4 70+								
<8	2134	85%	1604	82%	531	71%	4269	82%
≥8	161	6%	146	7%	75	10%	382	7%
Missing	228	9%	207	11%	143	19%	578	11%
MoCA at HUNT4 70+								
1–21	176	7%	463	24%	389	52%	1028	20%
22–30	2347	93%	1494	76%	356	48%	4197	80%
Missing	0	0%	0	0%	4	1%	4	0%
SPPB at HUNT4 70+								
0–8	289	11%	309	16%	245	33%	843	16%
9–12	2218	88%	1627	83%	488	65%	4333	83%
Missing	16	1%	21	1%	16	2%	53	1%

HUNT 4 70+: The Trøndelag Health Study, fourth wave, of people aged 70+; AiT: Ageing in Trøndelag study; MCI: Mild Cognitive Impairment; MoCA: Montreal Cognitive Assessment; HADS-D: Hospital Anxiety and Depression Scale—Depression; SPPB: Short Physical Performance Battery

The number of incident dementia cases among the 5229 baseline dementia-free population over the 4.2-year follow-up period was 749 (Table 1), hence, a cumulative dementia incidence proportion of 14.3% (749/5229) (14.0% in men and 14.6% in women). The incidence proportion increased with age. Among men, the incidence proportion was 13.3% for those aged 75–79 at baseline, 22.3% of those aged 80–84, and 43.8% of those aged 85 + . For women, the corresponding incidence proportions were 14.2%, 23.3%, and 48.9%, respectively (Table 2).

**Table 2.** Number and % changing status from dementia free at HUNT4 70+ to having dementia at follow-up in AiT.

Age	Dementia free at HUNT4 70+, n	Dementia at follow up in AiT, n	% dementia at follow-up in AiT	Crude dementia incidence rate
Men				
70–74	1293	127	9.8%	24.6
75–79	712	95	13.3%	34.1
80–84	327	73	22.3%	60.1
85–89	83	36	43.3%	135.4
90+	22	10	45.5%	144.3
All ages	2437	341	14.0%	35.9
Women				
70–74	1414	117	8.3%	20.6
75–79	831	118	14.2%	36.5
80–84	369	86	23.3%	63.2
85–89	145	67	46.2%	147.6
90+	33	20	60.6%	221.8
All ages	2792	408	14.6%	37.6
All ages, men and women combined	5229	749	14.3%	36.8

HUNT 4 70+: The Trøndelag Health Study, fourth wave, of people aged 70+; AiT: Ageing in Trøndelag study

Total person time was 20,353 person years (py). The crude dementia incidence rate was 36.8 per 1000 py (749/20,353), 35.9 in men and 37.6 in women (Table 2).

For those progressing to dementia at follow-up, 33.8% had no cognitive impairment at baseline, while the rest had MCI (55.8% had aMCI and 10.3% had naMCI) at baseline (Table 3). Among those with no cognitive impairment at baseline, 61.2% did not progress, while 31.2% progressed to MCI, and 7.6% progressed to dementia. Among individuals with MCI at baseline, 25.5% experienced a reversal to no cognitive impairment, 48.2% remained classified as having MCI, and 26.2% progressed to dementia during the follow-up period. The progression rate to dementia was 25.9% for individuals with aMCI and 28.0% for those with naMCI.

**Table 3.** Cognitive status at HUNT4 70+ and cognitive status at follow-up (row %). N = 5229.

	Cognitive status at HUNT4 70+:				
Cognitive status at follow-up at AiT:	No cognitive impairment	aMCI	naMCI	Other cognitive impairment	Total
No cognitive impairment	2040 (80.9)	409 (16.2)	74 (2.9)	0 (0)	2523 (100)
aMCI	812 (51.4)	688 (43.6)	78 (4.9)	1 (0)	1579 (100)
naMCI	228 (61.1)	102 (27.4)	43 (11.5)	0 (0)	373 (100)
Dementia	253 (33.8)	418 (55.8)	77 (10.3)	1 (0)	749 (100)
Other cognitive impairment	-	-	-	-	5 (100)
Total	-	-	-	-	5229 (100)

AiT: Ageing in Trøndelag study; aMCI: Amnestic Mild Cognitive Impairment; naMCI: Non-Amnestic Mild Cognitive Impairment.

Weighted for non-response between the two study waves, the overall dementia incidence rate per 1000 py for the 70+ population was 48.0 (95% confidence interval (CI) 44.3, 51.7), 43.7 in men and 51.7 in women (Table 4). Standardized to the Norwegian population in 2023 on age, sex and educ ation, the rate was 43.9 (95% CI 40.8, 47.1), and it was 42.5 in men and 45.2 in women.

**Table 4.** Dementia incidence (per 1000 py), by age and sex. Incidence rates are weighted using A) IPW to correct for non-response in AiT and B) IPW to correct for non-response in AiT and standardized to the dementia free Norwegian population in 2023 on age, sex and education.

Age		A. Weighted incidence rate per 1000 person years	95%CI Lower	95% CI Upper		B. Weighted and standardized incidence Rate per 1000 person years	95%CI Lower	95% CI Upper
Men								
70–74		27.9	21.9	33.8		28.8	22.7	35.0
75–79		30.2	24.3	36.1		31.6	25.4	37.9
80–84		61.3	49.0	73.5		62.6	49.8	75.4
85–89		86.4	59.7	113.2		87.2	61.2	113.2
90+		183.1	106.1	260.0		185.6	110.0	261.2
70+		43.7	38.9	48.6		42.5	37.9	47.0
Women								
70–74		18.9	14.3	23.6		18.4	13.9	22.9
75–79		35.3	28.5	42.1		34.0	27.7	40.2
80–84		62.8	50.4	75.1		58.0	46.8	69.2
85–89		119.6	92.8	146.4		120.5	94.0	147.0
90+		212.5	145.7	279.3		208.3	146.2	270.4
70+		51.7	46.2	57.3		45.2	40.9	49.6
Total								
70–74		23.3	19.6	27.1		23.5	19.7	27.3
75–79		32.9	28.3	37.5		32.8	28.4	37.3
80–84		62.1	53.3	70.8		60.1	51.6	68.5
85–89		106.2	86.9	125.5		107.1	88.2	126.1
90+		202.3	151.3	253.3		201.0	152.5	249.5
70+		48.0	44.3	51.7		43.9	40.8	47.1

AiT: Ageing in Trøndelag study; IPW: Inverse Probability Weighting; CI: Confidence Interval; py: person years.

For comparative purposes we applied the European standard population and various age ranges. Using the European standard population in 5-year age bands, and weighting for non-response, the dementia incidence per 1000 py was 42.5 for age 70–89 years, and 72.1 for age 75+ (Supplemental Table 1). Applying 10-year age weights for ages 70–99, the European age-standardized rate was 53.6 per 1000 py. In addition, in a sensitivity analysis without nursing home residents and home visits (with IPW) using the European standard population in 5-year age bands for 70–89 years, the dementia incidence was 37.3 per 1000 py (32.5 per 1000 py without IPW) (Supplemental Table 1).

For men, non-response weighted standardized (Norwegian standard population) dementia incidence rates per 1000 person years (py) increased from 28.8 at age 70–74 years, to 31.6 at 75–79 years, 62.6 for 80–84 years, 87.2 for 85–89 years and 185.6 at 90 + years (Table 4). For women the corresponding numbers were 18.4, 34.0, 58.0 120.5 and 208.3.

The number of incident dementia cases in the 70+ population in 2023 was estimated to be 28,937 using the IPW weighted incidence rates by age and sex, combined with the dementia free population size in Norway for year 2023 (Table 5). If we used the crude dementia incidence, without weighting for non-response and without standardization, the estimate was 24,885. By applying, and fixating, the same set of weighted and standardized incidence rates on population projections from Statistics Norway (subtracting the estimated dementia prevalence at the start of the year), the number of incident dementia cases for year 2030, 2040 and 2050, would be 35,474, 46,820, and 56,326, respectively (Table 5).

**Table 5.** Projections of number of new dementia cases in Norway towards 2050.

	Dementia free population in Norway^a^					Dementia incidence rates per 1000 py^b^			Incident dementia cases, based on crude incidence^c^					Incident dementia cases, based on weighted and std. incidence^c^			
Age, years	2023	2030	2040	2050		Crude rate	Weighted and std. rate		2023	2030	2040	2050		2023	2030	2040	2050
Men																	
70–74	118,014	128,710	153,583	144,291		25.7	28.8		3033	3308	3947	3708		3399	3707	4423	4156
75–79	97,054	101,751	120,588	137,850		27.1	31.6		2630	2757	3268	3736		3067	3215	3811	4356
80–84	48,621	74,383	85,289	106,986		54.2	62.6		2635	4032	4623	5799		3044	4656	5339	6697
85–89	20,542	30,869	45,600	58,939		61.6	87.2		1265	1902	2809	3631		1791	2692	3976	5139
90+	8418	10,426	21,991	31,021		163.9	185.6		1380	1709	3604	5084		1562	1935	4082	5757
Women																	
70–74	123,963	134,797	157,415	150,144		18.0	18.4		2231	2426	2833	2703		2281	2480	2896	2763
75–79	106,833	110,736	126,454	144,733		29.1	34.0		3109	3222	3680	4212		3632	3765	4299	4921
80–84	59,529	84,997	94,606	114,448		51.7	58.0		3078	4394	4891	5917		3453	4930	5487	6638
85–89	28,966	38,672	53,729	65,426		94.2	120.5		2729	3643	5061	6163		3490	4660	6474	7884
90+	15,449	16,481	28,958	38,476		180.9	208.3		2795	2981	5239	6960		3218	3433	6032	8015
																	
Total (men and women 70+)	627,388	731,823	888,213	992,314		36.7	43.9		24,885	30,375	39,955	47,912		28,937	35,474	46,820	56,326

^a^ The dementia free population sizes in Norway at the start of the year for years 2023, 2030, 2040, 2050 was estimated using population data from Statistics Norway Stat bank, subtracting the estimated prevalence of dementia at the start of the year using age- and sex specific dementia prevalence estimate^3^ (dementia prevalence for men at ages age 70–74, 75–79, 80–84, 85–89, 90+ were 6.4%, 10.0%, 17.8%, 30.4%, and 41.5%, respectively. For women, the corresponding numbers were 4.8%, 9.0%, 18.0%, 34.6% and 50.9%).

^b^ Then, two sets of incidence rates were calculated; 1. Crude, 2. Weighted for non-response and standardized to the Norwegian population aged 70+ in 2023 by age, sex and education (primary, secondary, tertiary). All rates were calculated allowing for changing age-groups during follow-up for dementia free individuals (Lexis). Thus, crude rates do not correspond to those in Table 2, which had no such Lexis approach.

^c^ Incident dementia cases for the current year 2023 were estimated by multiplying the dementia free population with the incidence rate. Projections of incident dementia cases for years 2030, 2040, and 2050 were estimated the same way as for year 2023, but using population projections (main alternative).

## Discussion

In our longitudinal study of 70+ year adults followed over more than four years, the dementia incidence proportion was 14.3%, which corresponded to a standardized dementia incidence rate of 43.9 per 1000 person years, slightly higher in women than in men. The dementia incidence increased sharply with age, with roughly a doubling every fifth year. Based on these results, the projected number of incident dementia cases in Norway is expected to nearly double from around 29,000 in 2023 to 56,000 by 2050.

To compare our results with other studies, we used age-specific dementia incidence rates for ages 70–89 from Mukadam et al.^11^ and Wolters et al.,^8^ calculated age-standardized rates using the direct method and the European standard population. These rates were: PAQUID (Personnes Agées QUID), 27.2, Rotterdam Study 41.1, Framingham Heart Study 24.5, CFAS (Cognitive Function and Ageing Study) II 19.0, Three-City Study 16.1, AGES-Reykjavik 22.2, Einstein Ageing Study 15.0,^28^ while in our study the standardized rate, using the same standard population and age groups was 42.5 (Supplement Table 1). A Swedish study, using data from two cohorts, the Kungsholmen Project (KP) and the Swedish National study on Aging and Care in Kungsholmen (SNAC-K), reported dementia incidence for ages 75+ (75–79, 80–84, 85–89, 90+). The age standardized rates for these were 72.3 for the KP cohort 1987–89, and 52.4 for the SNAC-K cohort 2001–4.^7^ Using same age groups and standard population, our estimate was 72.1. Finally, a recent Norwegian study with overlapping time period as in our study,^12^ published results for 10-year age bands. Applying age bands 70–79, 80–89, and 90–99, the European age-standardized rate for Tromsø7 (2015–19) was 17.1, while for our study it was 53.6. Thus, the Tromsø7 dementia incidence rate was only one third of our rate. This might reflect the lower sensitivity in studies relying on register-based dementia diagnoses. Our study used a clinically based dementia diagnosis with higher sensitivity. Hospital-based data might miss a significant portion of the dementia population, underestimating the true incidence. This aligns with Gjøra et al.,^29^ who found that approximately one-third (35.6%) of the HUNT4 70+ respondents with research dementia diagnosis had hospital dementia diagnosis. Furthermore, the Tromsø study excluded home visits resulting in a healthier population compared to HUNT. This is reflected in the low frailty estimates of 3.8% in the 70+ population in Tromsø5 versus 10.6% in HUNT4, both using Fried's frailty criteria.^30,31^

Thus, our dementia incidence estimates were higher than most other comparable studies, except from the Dutch Rotterdam study,^32^ the Swedish KP study and the Swedish SNACK study.^7^

The higher estimates in our study may be attributed to several factors. Trøndelag may have a higher incidence rate of dementia, potentially due to a frailer population, and HUNT's inclusion of nursing home residents and home interviews reduces healthy selection bias.

Another plausible explanation lies in differences in study designs, diagnostic procedures, as well as in methods of analysis and weighting. A further explanation is the use of different diagnostic criteria. We used DSM-5, which can capture more cases than the ICD-10 criteria.^33^ Notably, several cited studies were conducted before the fifth edition of the DSM criteria and relied on earlier editions, which could result in varying incidence rates and classifications of neurocognitive disorders compared to our findings. Several factors contribute to the higher incidence rates observed in the AiT study compared to other studies. HUNT4 70+ and AiT utilizes detailed clinical assessments, home visits, and next-of-kin interviews. This level of thoroughness likely results in higher detection rates of dementia. Data collection in field stations, homes, and nursing homes allows capturing a broader demographic, including individuals with advanced cognitive decline who might be overlooked in other studies. To the latter, we noted that without IPW, our European age standardized estimate from age 70–89 was reduced from 42.5 to 35.9, and by exclusion of nursing home patients and frail people assessed at home (IPW was re-applied), the rate was further reduced to 32.5 per 1000 py. Thus, selection might affect dementia incidence to a large degree, and failing to adjust for this might bias results downwards.

The Rotterdam Study, like PEQUID, Gothenburg Study, Three-City, CFAS II, Framingham, AGES-Reykjavik, and Einstein Aging Study, used a consensus panel to agree on a cognitive diagnosis, like our approach. However, the SNACK Study did not. All studies except the Einstein Aging Study included participants in nursing homes. Focusing on those aged 70+, AiT observes higher dementia incidence due to increased age-related risk.

Through a rigorous methodology incorporating clinical assessments, field station evaluations, home visits, and next-of-kin interviews, our study ensures comprehensive data collection on cognitive decline and dementia, mirroring the approaches of well-regarded studies like the Rotterdam Study. HUNT4 70+ and AiT provide robust incidence rate data, supported by meticulous methods like the MoCA scale and CERAD Word List Memory Task, comparable to the rigorous protocols of CFAS, PAQUID, and Three-City. Similarly, the Rotterdam and Gothenburg Studies employ stringent assessments, contributing to their reliability. Unlike HUNT, AGES-Reykjavik also integrates genetic data, adding another layer of complexity to their findings.

Our findings that late-life depression may predict dementia are supported by several studies.^34–37^ However, the Lancet Commission on Dementia classifies depression as a midlife risk factor, noting some late-life associations may stem from preclinical dementia.^37^

Several studies have reported a decrease in dementia incidence for those aged 60 and above, such as a 20% decline per decade in the Framingham Heart Study, a 30% decrease in the Gothenburg studies, and a 20% reduction noted in the UK's CFAS I and II. Although our study did not examine incidence trends over time, we observed higher incidence rates in the HUNT4 cohort compared to these studies. This discrepancy may be due to regional or methodological differences. Although some research suggests that factors like rising obesity, sedentary lifestyles, and improved survival rates from stroke and type II diabetes could reverse the recent decline in dementia incidence,^38^ we have not investigated how these factors might explain the rates found in our study. Methodological differences can challenge the comparability of results across cohorts. Nevertheless, disparities in incidence rates between our study and others underscore the importance of conducting rigorous and inclusive data collection, as well as comprehensive follow-up.

### Strengths and limitations

The study benefits from the representative nature of the HUNT4 population-based sample, accompanied by thorough data collection protocols, robust infrastructure, and expert-led diagnostic categorization. It adheres to recognized quality standards for dementia incidence research,^8,39^ featuring a diverse and extensive sample that spans from normal cognition to severe dementia. The entire population of a Norwegian county, including home and institution residents, was invited. Standardized methods and validated criteria were used in a one-phase design with cognitive tests, disability assessments, informant and clinical interviews. The response rate was just over 50% in HUNT4 70+ and slightly over 70% in AiT, aligning with recent studies.^11^ Non-participants likely differed in variables such as age, sex, and education. Therefore, we weighted our analyses for non-response bias and calibrated the results to accurately represent Norway's age, sex, and education distribution. Multiple assessors, all trained health personnel, conducted the data collection to ensure high reliability. Most studies on dementia prevalence use either algorithmic or clinical consensus diagnostic methods.^11^ We selected the clinical consensus method to reflect current diagnostic practices and include comprehensive information. While it may be considered less reliable than algorithmic approaches due to its reliance on subjective expert judgment, which can introduce variability and bias, it nonetheless leveraged the collective expertise of our expert group to standardize interpretations.^40^ Limitations include reliance on secondary information and absence of biomarker data. Additionally, the analysis is based on only two time points and four years of follow-up, which may limit our estimates of dementia incidence over longer periods. Caution is needed when applying results to other countries due to varying population characteristics, healthcare systems, and diagnostic practices.

### Conclusion

Incidence rates in this large population-based Norwegian study using clinical dementia assessment, were at the higher end, and much higher than previous registry-based estimates from Norway, but comparable to results from Sweden and the Netherlands. In this study we have provided reliable and precise incidence estimates of dementia applying a population-based sample of individuals aged 70+, using data from the HUNT Study. The findings of this study highlight the current burden of dementia. While we have projected the expected rates of dementia within the aging population, further research is necessary to determine whether these incidence rates are increasing over time. Our findings have significant implications for healthcare planning, early detection strategies, and interventions aimed at promoting cognitive health and improving the quality of life for older adults. By identifying current and future trends in cognitive impairment, policymakers and healthcare providers can better develop targeted interventions to address growing public health challenge.

## Supplemental Material

sj-docx-1-alz-10.1177_13872877251371242 - Supplemental material for Incidence of dementia among individuals 70 years and older in Norway: A HUNT study Supplemental material, sj-docx-1-alz-10.1177_13872877251371242 for Incidence of dementia among individuals 70 years and older in Norway: A HUNT study by Inger Molvik, Bjørn Heine Strand, Anne Marie Mork Rokstad, Eivind Aakhus, Stina Aam, Sverre Bergh, Anne Brækhus, Knut Engedal, Linda Gjøra, Grete Kjelvik, Marte Kvello-Alme, Gill Livingston, Fiona E Matthews, Karin Persson, Håvard Kjesbu Skjellegrind and Geir Selbæk in Journal of Alzheimer's Disease
