# Supplementary material for: Incidence of dementia among individuals 70 years and older in Norway: A HUNT study
Source: J Alzheimers Dis. 2025 Aug 29;107(4):1469–80. doi: 10.1177/13872877251371242 (PMC12678644; doi:10.1177/13872877251371242)
Supplement: sj-docx-1-alz-10.1177_13872877251371242 - Supplemental material for Incidence of dementia among individuals 70 years and older in Norway: A HUNT study [file sj-docx-1-alz-10.1177_13872877251371242.docx]

# **Supplemental Material**

# **Incidence of dementia among individuals 70 years and older in Norway: A HUNT study**

# **Supplemental Table 1.** Age standardized dementia incidence rates (per 1000 persons), European standard population. Rates for external studies collected from Table 2 in <https://www.neurology.org/doi/10.1212/WNL.0000000000010022>

| Age years | European standard population | HUNT4 70+ | PAQUID | Rotterdam Study | Framingham Heart Study | Gothenburg studies | CFASII | Three-City Study | AGES-Reykjavik | HUNT4 70+ without IPW weighting  (sensitivity analysis 1) | HUNT4 70+, nursing home and home visits excluded (with IPW)  (sensitivity analysis 2) | HUNT4 70+, nursing home and home visits excluded (without IPW)  (sensitivity analysis 3) |
| --- | --- | --- | --- | --- | --- | --- | --- | --- | --- | --- | --- | --- |
| 70-74 | 5000 | 23.3 | 5.9 | 19.5 | 9.7 | 8.0 | 8.2 | 6.3 | 7.9 | 21.7 | 22.5 | 20.9 |
| 75-79 | 5000 | 32.9 | 26.5 | 37.2 | 17.9 | 18.6 | 16.4 | 12.8 | 15.7 | 28.2 | 30.0 | 26.6 |
| 80-84 | 2500 | 62.1 | 43.6 | 58.3 | 41.0 | 43.2 | 32.1 | 23.1 | 37.3 | 52.9 | 52.7 | 46.4 |
| 85-89 | 1500 | 106.2 | 73.1 | 97.0 | 67.9 | 73.3 | 42.2 | 48.2 | 66.3 | 80.1 | 85.7 | 67.4 |
| **Age-standardized 70-89** |  | **42.5** | **27.2** | **41.1** | **24.5** | **25.1** | **19.0** | **16.1** | **22.2** | **35.9** | **37.3** | **32.5** |
|  |  |  |  |  |  |  |  |  |  |  |  |  |
| Age, years | European standard population | HUNT4 70+ | Tromsø7 |  |  |  |  |  |  |  |  |  |
| 70-79 | 9000 | 28.6 | 7.7 |  |  |  |  |  |  |  |  |  |
| 80-89 | 4000 | 76.3 | 30.1 |  |  |  |  |  |  |  |  |  |
| 90-99 | 900 | 202.3 | 52.8 |  |  |  |  |  |  |  |  |  |
| **Age-standardized 70-99** |  | **53.6** | **17.1** |  |  |  |  |  |  |  |  |  |
|  |  |  |  |  |  |  |  |  |  |  |  |  |
| Age, years | European standard population | KP Cohort | SNAC-K cohort | HUNT4 70+ |  |  |  |  |  |  |  |  |
| 75-79 | 4000 | 45.2 | 27 | 32.9 |  |  |  |  |  |  |  |  |
| 80-84 | 2500 | 81.9 | 56.6 | 62.1 |  |  |  |  |  |  |  |  |
| 85-89 | 1500 | 108.1 | 70.3 | 106.2 |  |  |  |  |  |  |  |  |
| 90+ | 1000 | 103.0 | 116.4 | 202.3 |  |  |  |  |  |  |  |  |
| **Age-standardized 75+** |  | **72.3** | **52.4** | **72.1** |  |  |  |  |  |  |  |  |
